# Fostering change, empowering faculty: comments on the NURSLITT study and the five-year rule

**DOI:** 10.5195/jmla.2024.1768

**Published:** 2024-04-01

**Authors:** Eleanor Shanklin Truex, Jean Hillyer, Emily N. Spinner

**Affiliations:** 1 etruex62@gmail.com, Ascension Saint Joseph Hospital; 2 jean_hillyer@baylor.edu; 3 emily.spinner@gmail.com, Ellis Hospital

**Keywords:** 5-year Rule, Date Limits, Date Range, Literature Searches, Search Limits, Nurses, Nursing Faculty, Nursing Education, Nursing Research

## Abstract

The five-year rule must die. Despite an extensive literature search, the origins of the five-year rule remain unknown. In an era when the nursing profession is so focused on evidence-based practice, any approach that arbitrarily limits literature searches to articles published in the previous five years lacks scientific basis. We explore some reasons for the pervasiveness of the practice and suggest that librarians need to engage with nursing faculty, who are well-positioned to be change agents in this practice.

Experienced librarians know the search habits of their patrons are as varied as the people they assist. One such idiosyncrasy is the persistent practice of “the five-year rule” search limit within the nursing profession. During our recent study on this topic, members of our research team noted a few not readily apparent influences that may have some bearing on this entrenched habit. Our objective is to deepen librarians' understanding of the five-year phenomenon with a view toward fostering change.

“The five-year rule” is the stipulation that references used within a paper (if a student) or a publication (if professional) be no older than five years from the date of the assignment or piece. Health sciences librarians have long objected to the stringent date ranges imposed by nursing faculty upon their students for nursing literature searching; the subsequent persistence of this “rule” after entering their professional nursing careers remains a concern.

Despite an exhaustive literature search, the only sources found that discuss this limitation were a few editorials written by nursing professionals decrying this very practice. In our study (Truex et al., 2022) we interviewed nursing students, faculty, direct care nurses, and health sciences librarians to assess their thoughts on literature searching practices by those in nursing. In general, all nursing participants (regardless of status) viewed the five-year rule favorably; only the librarians were tempered in their evaluation of its application [1]. Reasons posited by faculty for the use of the five-year rule included: it provides boundaries and structure, variations on “this is what I learned, so this is what I teach,” five years is ideal because nursing skills change and evolve, and remarkable claims like “if we said 10 years, [the students] would find twice as many articles.” [1]. Five years can be an appropriate date range for some topics, but that context is key: selecting appropriate date ranges for literature searches is entirely topic-dependent.

Through these focus groups, the elements that coalesce to perpetuate this practice became apparent. Given human behavior, there are no absolutes, but we believe awareness of these elements will assist librarians to foster change. Three factors appear to be involved in this tenacious convention: research literacy, the human response to task complexity, and the characteristics of innovation that affect its diffusion rate.

## RESEARCH LITERACY?

The factors that influence the information searching behaviors of nursing students, faculty, and practicing clinicians are complex. Nursing is a relatively young scientific discipline. It has been using modern research methods only since the 1980s and 1990s, and the responses to those studies, as well as the replication and furtherance of them, is still ongoing [2]. Given nursing research's relative youth, research literacy is vital. Beaudry and Miller define research literacy as: “… the ability to locate, understand, discuss and evaluate different types of research; to communicate accurately about them; and to use findings for academic and professional purposes” [3].

Research literacy facilitates the ability to assess, plan, implement, and evaluate gaps in process or knowledge across the profession: the essence of nursing. However, it is an overarching concept addressing competency, not a process to achieve that intention. General consensus within the literature advocates increasing nursing students' and practicing nurses' research literacy [4] via differing pedagogical approaches [5, 6]. Hypothesis or PICO development, for which the review of the literature is a crucial element, is rightly emphasized in nursing pedagogy. It is the tactics currently used for the literature exploration portion that can hamper the nursing research process with a subsequent deficiency in research literacy. The practice of instructing nursing students to use stringent date limits when searching the nursing literature is standard for many undergraduate nursing programs. It is a “rule” of long standing: one of the authors was taught it in her BSN program in the 1980s. Medical librarians in our study shared comments such as “[A nursing assignment reference] cannot be 5 years and one month old.”

It can be difficult to find something that is not there. In reviewing a range of nursing research textbooks (1959-2020), along with a variety of chapters dealing with reviewing the literature that were sent to us via colleagues subscribed to the MEDLIB-L mailing list, we found no evidence to support the use of the five-year time frame when conducting literature reviews for academic or research purposes. Burns and Grove (2009) sum up the general view held in these books: “Students repeatedly ask, ‘How many articles should I have? How far back in years should I go to find relevant information?’ The answer to both questions is an emphatic *‘It depends.'*” (emphasis ours) [7]. Other textbooks recommend 10 years or simply state that for fundamental works the year of publication should not be a concern. This is a far cry from the strict parameters taught in many nursing schools. In addition, the APA, the primary citation tool used in nursing schools, declares:

“Many writers incorrectly believe that sources cited in APA Style papers must have been published recently, such as within the last 5–10 years. That's a myth. There is no timeliness requirement in APA Style guidelines. We recommend citing reliable, primary sources with the most current information whenever possible. What it means to be “timely” varies across fields or disciplines.” [8]

It is not solely in research practice that the five-year rule can be problematic. The two primary accreditation bodies in nursing education, the American Association of Colleges of Nursing (AACN) and the Accreditation Commission for Education in Nursing (ACEN), both appear to reinforce this rule. The representatives we contacted at these accrediting bodies affirmed this. We were unable to determine if this date range is stipulated in the actual site visit standards or by the nursing faculty interpreting terms such as “current” to indicate no older than five years for reference material. This tenet is passed, not only through nurses, but among medical librarians. One of our authors relates that when she began her career, a veteran nursing school librarian advised preparing for an accreditation site visit by removing or discarding anything on the shelves older than five years, resulting in hundreds of books being thrown out. These sorts of collection development strategies built around the five-year rule have proliferated throughout the profession, despite the AACN's Commission on Collegiate Nursing Education not stipulating a date range: “The 2018 CCNE Standards for Accreditation of Baccalaureate and Graduate Nursing Programs does not specify any type of timeframe for purging library materials” [9].

This emphasis on dismissing literature older than five years, along with the inadvertent reinforcement by institutional policy, affects nursing researchers. We found several articles positing that nursing information doubles every five years, all based on a source that provides no evidence or citations for this claim [10, 11, 12]. This limitation on five-year data has other effects as well, such as adding unnecessary (and limiting) bias into nursing evidence synthesis studies. For example, Lu et al. 2019's examination of nursing job satisfaction scrupulously limited their searches of ten unique databases to within five years [13], while DeVon's 2007 literature review methodology notes that, “Nursing research articles were eligible for inclusion if they were published in the last 5 years…” [14]. There are likely more such examples elsewhere in the literature.

This disconnect between what is taught/practiced versus what is used in actual academic nursing research reveals an issue central to research literacy: effective searching of the available literature is required to foster skills needed for competency in understanding nursing research. Search skills as currently taught in nursing schools at the undergraduate level are inadequate. Sakalys in 1984 said, “A single, isolated intervention (i.e. a research course taught at the end of a nursing program) is not likely to promote development of cognitive processes fundamental to scientific inquiry” [15]. In speaking to the librarians in our focus groups, common practice appears that a basic introduction to EBP and research unit is given, but what is practiced is not the same as what is preached. Schuessler echoed Sakalys' stance, stating that the basic introduction to EBP and research may be the nurses first (and only) exposure to this information, adding that when nurses are in need of information, their preference is to ask colleagues rather than conduct a literature search, and emphasized, “Nurse participants from these studies believe that patient care should be based on research but lacked the skills, comfort, and resources to access, appraise, and implement research” [16].

## HUMAN RESPONSE TO TASK COMPLEXITY

Human behavior and response, coupled with the need for appropriate applied searching skills, must be factored into research pedagogy, our second factor at play. Bystrom found that increasingly complex information acquisition makes it more likely that people will turn to other people as sources, rather than to documentary sources [17]. Wakeham elaborates on this issue while speaking to the librarian's role. Nurses rely on colleagues when seeking complex information related to their practice, which is problematic because there is no quality control. Wakeham goes on to say, “The librarian has a contribution to make here…. They could achieve a great deal by becoming more skilled themselves in personally imparting information to the user, and in making themselves more prominent in [their] environment…” [18].

Despite no published evidence for the five-year rule in the decades we reviewed, it is still taught in class lectures, and reinforced via assignment parameters. Furthermore, when faced with the complex task of searching nursing literature, nursing students appear to rely on their instructors' guidance rather than what their course readings recommend. Burns & Grove alluded to this: “When writing a course paper…clarify with your professor the publication years and type to be included” [19], unknowingly reiterating the Bystrom and Schuessler's contentions regarding people vs. documentary sources. This inclination hampers research literacy as nurses self-limit, decreasing their awareness of relevant resources and the context of the accessed material. When these same nurses later ask a work colleague for suggestions on searching date ranges, it is likely that five years or fewer will be recommended, perpetuating the practice.

This commentary is not a how-to guide for librarians; there is no specific strategy that will guarantee the use of topic-dependent date limits by nursing personnel, but librarians are not without agency to end the five-year rule dominance. Dismantling this ubiquitous “standard” will take time, but if presented with the facts and rationales as laid out here, nursing pedagogy should embrace this change. In the presentations by the authors made to groups of nursing faculty, they have been receptive to considering discontinuing the use of the five-year rule in nursing education.

## DIFFUSION OF INNOVATIONS

The third and final factor, diffusing innovations, is useful to foment change. Everett Roger's theory of “diffusion of innovation” is applicable to the five-year rule shibboleth. The theory posits five characteristics of innovations that influence the rate at which they are adopted. To be rapidly adopted, an innovation must be perceived as being advantageous, compatible with existing values, easy to understand and use, “trial-able,” and visible to others [20]. We apply it here both to nurses in academic and direct care settings, and to the librarians who serve them. The five-year rule easily meets all these criteria: it's certainly “trial-able”—the results are observable immediately. It's our opinion that dropping the use of strict date limits hinges on considering Roger's first two characteristics. The first characteristic is likely the stickiest: a narrow five-year window may be perceived as providing a relative advantage over broader ranges for limiting literature searches, given that novice researchers feel inundated by data and information. Also, the five-year rule can be seen by novice nurses as aligning with the values of their nursing faculty and mentors. This perceived alignment is especially important, as nurses weigh the models provided by their instructors and mentors more heavily than recommendations in print sources, a weighting that is further reinforced at the institutional level via accreditation standards.

## CONCLUSIONS AND RECOMMENDATIONS FOR LIBRARIANS

Our proposal for medical librarians: adapt this “diffusion of innovation” to promote appropriate use of date limits among nurses at all levels. We should also address the need for such “innovation” from more than one angle; by educating nursing students, faculty, and direct care nurses, and we should also use this diffusion of innovation to persuade. Librarianship is a profession that sits at the hub of many others, a position favorable to truly make a difference in the healthcare sciences, but a more concerted effort than commentary or concern amongst ourselves is needed. Librarians should take initiative by contacting nursing faculty with their concerns prior to the start of the academic year. Within our research guides and other digital learning offerings, librarians should provide rationales on how to think critically during literature searches to discourage the use of inappropriately applied date limits. Librarians should petition to be included on the committees that work toward nursing school accreditation and educate those members on the importance of broader date ranges for reference material and literature searching. Library orientations during new faculty onboarding should discourage use of stringent date restrictions. Sharing Op-Ed content such as the following may help faculty alter their view:

“Our pioneers' names are not on reference lists because we have the absolutely stupid 5-year-rule in nursing! Students are told that their references are to be no older than 5 years. Why? Because some well-meaning but narrow individuals[sic] decided that nursing is a “science”; therefore, only recent publications need apply (in truth, it's an art and a science). As my colleague and associate editor Dr. Eleanor Covan points out, “Even in the so-called ‘hard' sciences there is no such rule; if the research is about telescopes, Galileo[sic] is always cited.” [21]

Librarians can point to our original research and this commentary, or check within their own collections' nursing research textbooks, if they feel the need to cite their sources. In the clinical setting, librarians should educate staff on the use of pertinent date restrictions (if applicable) for references in hospital policies, quality improvement projects, and unit-specific evidence-based practice pilots. Once a sufficient number of “early adopters” and “power brokers” within the faculty or clinical nursing leadership are persuaded to make this change, the judicious use of date ranges will be seen as advantageous for all. If people are the preferred source for expert knowledge, librarians certainly qualify. Individual situations vary, but a persuasive approach, rather than a simply factual one, should prove more successful. The nursing groups our research team has spoken to since publication have all welcomed this discussion. Persistent and consistent messaging from librarians will foster acceptance and change among faculty and clinical nurses.

We cannot state with certainty that the persistent application of the five-year rule to nursing literature searches negatively impacts nursing research literacy, but neither can it be said that relying on any rigid time frame fosters progress in nursing science. Common sense dictates that circumstances alter cases. One new trend noted in our updated literature review for this commentary was to find that many nurse authors now include “no date limits were used” in their abstracts. We hope this trend of delineating whether date ranges were imposed becomes a standard; librarians know that too many nursing projects do not start this way.

Unless librarians initiate the appropriate use of date limits in nursing literature searches, progress will be nonexistent, given the human response to task complexity and innovation. In fact, the status quo has worked for the nursing profession so far, so it's unlikely they will change this practice on their own. We hope that with a deeper understanding of the factors at play, health science librarians will feel more resolute and empowered to foster critical thinking in literature searching among their nursing colleagues at all levels.
